# Maternal and Neonatal Polyunsaturated Fatty Acid Intake and Risk of Neurodevelopmental Impairment in Premature Infants

**DOI:** 10.3390/ijms23020700

**Published:** 2022-01-09

**Authors:** Rory J. Heath, Susanna Klevebro, Thomas R. Wood

**Affiliations:** 1 Emergency Medicine Department, Derriford Hospital, University Hospitals Plymouth NHS Foundation Trust, Plymouth PL68DH, UK; rory.heath2@nhs.net; 2 Department of Clinical Science and Education, Södersjukhuset, Karolinska Institutet, 11883 Stockholm, Sweden; susanna.klevebro@ki.se; 3 Department of Pediatrics, University of Washington, Seattle, WA 98195, USA; 4 Center on Human Development and Disability, University of Washington, Seattle, WA 98195, USA; 5 Institute for Human and Machine Cognition, Pensacola, FL 32502, USA

**Keywords:** polyunsaturated fatty acid, fatty acid, docosahexaenoic acid, arachidonic acid, brain, infant, pregnancy, premature birth, preterm infant, premature infant, neurodevelopment

## Abstract

The N3 and N6 long chain polyunsaturated fatty acids (LCPUFA) docosahexaenoic acid (DHA) and arachidonic acid (AA) are essential for proper neurodevelopment in early life. These fatty acids are passed from mother to infant via the placenta, accreting into fetal tissues such as brain and adipose tissue. Placental transfer of LCPUFA is highest in the final trimester, but this transfer is abruptly severed with premature birth. As such, efforts have been made to supplement the post-natal feed of premature infants with LCPUFA to improve neurodevelopmental outcomes. This narrative review analyzes the current body of evidence pertinent to neurodevelopmental outcomes after LCPUFA supplementation in prematurely born infants, which was identified via the reference lists of systematic and narrative reviews and PubMed search engine results. This review finds that, while the evidence is weakened by heterogeneity, it may be seen that feed comprising 0.3% DHA and 0.6% AA is associated with more positive neurodevelopmental outcomes than LCPUFA-deplete feed. While no new RCTs have been performed since the most recent Cochrane meta-analysis in 2016, this narrative review provides a wider commentary; the wider effects of LCPUFA supplementation in prematurely born infants, the physiology of LCPUFA accretion into preterm tissues, and the physiological effects of LCPUFA that affect neurodevelopment. We also discuss the roles of maternal LCPUFA status as a modifiable factor affecting the risk of preterm birth and infant neurodevelopmental outcomes. To better understand the role of LCPUFAs in infant neurodevelopment, future study designs must consider absolute and relative availabilities of all LCPUFA species and incorporate the LCPUFA status of both mother and infant in pre- and postnatal periods.

## 1. Introduction

The N3 and N6 long-chain polyunsaturated fatty acid (LCPUFA) families comprise fatty acids with double carbon bonds at the N3 or N6 positions, respectively. The N3 family includes alpha-linolenic acid (ALA), eicosapentaenoic acid (EPA), and docosahexaenoic acid (DHA), while important N6 members include linoleic acid (LA) and arachidonic acid (AA). Humans cannot synthesise ALA and LA de novo, thus these LCPUFA are termed ‘dietarily essential’. Some conversion within the series of N3 or N6 fatty acids occurs endogenously, thus DHA and AA can be synthesized from their respective precursors. Synthesis occurs via enzymatic elongation and desaturation by 5- and 6-desaturase enzymes, but the efficiency of this process may be affected by genetic polymorphisms of the FADS gene [1]. While infants express these enzymes in utero, the capacity to synthesise AA and DHA from their precursors is inadequate to meet the LCPUFA demands of rapid infant development and growth [2,3,4,5]. Therefore, infants are dependent on preformed AA and DHA supplied by the placenta in utero, by breastmilk in the postnatal period, or by exogenous nutrition after preterm birth.

This review aims to explore the current evidence surrounding LCPUFA supplementation to improve neurodevelopmental outcomes after preterm birth. We discuss the surrounding context of LCPUFA: how these fatty acids accrete into fetal tissues during gestation and after birth and how the physical nature of these LCPUFA molecules and their subsequent metabolism into bioactive mediators exert their wide-ranging effects upon infant brain development. This context provides a base from which we may view the effects of changes to infant LCPUFA status through the current standard of care or through interventional trials (described in Supplementary Table S1), and it speculates upon guidance for practice and future research. Our scope extends to suggest that further clinical and research considerations may be needed to make LCPUFA provision in the preterm period more physiological for the infant, accounting for placental physiology, maternal LCPUFA status, and wider contexts of LCPUFA availability. 

This narrative review synthesizes evidence from articles identified in the reference lists of key preceding narrative and systematic reviews. New articles were identified via searches using the PubMed search engine of its constituent databases, MEDLINE and PubMed Central, and through active work in this research field amongst the authors. Keywords searched included ‘preterm/premature infant’, ‘polyunsaturated fatty acids’, ‘docosahexaenoic acid’, ‘arachidonic acid’, and ‘neurodevelopment’. A previous systematic review of the effects of LCPUFA supplementation in preterm infants has been performed by the Cochrane Collaboration in 2016 [6]. All papers that were analyzed systematically in the Cochrane review are included herein; a new systematic review is not warranted, as no new RCTs assessing the effects of LCPUFA supplementation upon neurodevelopment have been performed. Importantly, while excluded from the Cochrane systematic review due to not fulfilling RCT criteria, studies by Almaas et al. [7], Alshweki et al. [8], Collins et al. [9], Makrides et al. [10], and Smithers et al. [11] provide valuable wider insights into the field and are included in this narrative review. 

## 2. Why Are DHA and AA Important in the Developing Brain? 

### 2.1. LCPUFA and Neurodevelopment

DHA and AA together comprise a quarter of all brain fatty acids, particularly contributing to myelination and being enriched in neuronal synapses and cellular membranes [12], where their structure lends fluidity that aids neuronal functions such as neurotransmission [13,14].

Placental transfer and accretion of fatty acids into infant tissues is highest during the final trimester of pregnancy, with an essential reserve of AA and DHA stored in adipose in preparation for the post-natal demands of neurological development. Premature birth severs materno-fetal transfer, resulting in less LCPUFA availability relative to infants born at term, which may compromise neurodevelopment.

DHA is neuroprotective in animal models of neonatal asphyxia [15] and appears to encourage brain growth, myelination, and overall survival in prematurely born pigs [16]. In rats, improving LCPUFA availability to the offspring by supplementing the maternal diet can reduce the neurological damage associated with neonatal hypoxic-ischaemic brain injury [17]. Autopsy studies of human infants fed LCPUFA-deficient formula demonstrate the substitution of alternative N6 and N9 fatty acids in neural tissues [18,19]. 

DHA is also richly incorporated into photoreceptors, and deficiency is associated with reduced visual acuity and impaired visual transduction [20]. Randomized trials have shown reduced risk of severe retinopathy of prematurity (ROP) in preterm infants receiving enteral supplementation with DHA as well as a combination of DHA and AA [21,22]. ROP is a disease additionally associated with lower brain volumes and neurodevelopmental scores at two years of age [23]. Furthermore, greater erythrocyte levels of DHA correlate with positive MRI findings of brain microstructure development and with improved neurodevelopmental scores in infants born prematurely [24].

AA has multiple essential and wide-ranging roles in general infant development as well; dietary AA deficiency has been associated with growth impairment, which improves on return of dietary supply [25]. Feeding infants DHA unopposed by AA has also been associated with reduced preterm infant growth [25,26]. 

Overall, infants born prematurely and/or at a low-birth weight, thus with reduced adipose stores of LCPUFA, are more likely to experience neurosensory impairment, achieve lower levels of educational attainment, and have lower IQ scores [27]. 

### 2.2. LCPUFA Influence Inflammatory Signaling

AA exerts effects via its metabolism into bioactive molecules termed eicosanoids by the enzymes cyclooxygenase (COX) and lipoxygenase (LOX) [28]. Increased metabolism of AA into eicosanoids, such as prostaglandins, thromboxanes, and leukotrienes, occurs in response to infection or injury with consequent changes to vessel flow and permeability to aid delivery of immune cells [28]. For this reason, AA is often considered a pro-inflammatory agent. Conversely, DHA competes with AA for placement in cellular membranes and for metabolism by COX and LOX, and in doing so, it inhibits the production of eicosanoids and thus can be thought of as grossly having anti-inflammatory actions [28]. 

However, the roles of AA and DHA within inflammation are not binary; some AA-derived metabolites regulate the process of inflammation resolution. The prostaglandin PGE2 promotes the initial inflammatory response but subsequently acts to inhibit LOX to reduce further production of pro-inflammatory prostaglandins [29]. Some eicosanoids may protect and repair vessel membranes after injury [12]. Furthermore, PGE2 contributes to a ‘class switching’ event that stimulates COX and LOX to metabolize AA and DHA into lipid mediators such as lipoxins, resolvins, maresins, and neuroprotectins [29]. These metabolites, termed specialized pro-resolving mediators (SPM), inhibit inflammation and promote resolution to return tissues to pre-injury homeostasis [30,31]. While AA contributes to the production of lipoxins, the majority of SPMs are derived from DHA, including neuroprotectin D1 (NPD1), which has been shown to protect against oxidative stress and influence cell survival [32]. 

Although excessive inflammation is associated with negative outcomes [33], controlled inflammation is a necessary response to infection or injury. Imbalances in the N3:N6 ratio may therefore lead to domination of one fatty acid series over the other during competition for metabolism by COX and LOX, with consequent effects upon the availability of pro- or anti-inflammatory mediators and of pro-resolution agents.

While the balance of N3:N6 fatty acids have the potential to disrupt the inflammatory process, there is not yet a broad base of evidence to understand how the amounts and timing of LCPUFA administration may influence outcomes after premature birth. Within the available evidence, an observational study of LCPUFA blood levels of prematurely born infants associated low levels of DHA with an increased risk of chronic lung disease, while low levels of AA in the blood were associated with an increased risk of sepsis [34]. On the other hand, two large, randomized trials have failed to demonstrate a positive effect of postnatal DHA supplementation on preterm lung development [35,36]. The complexity of the AA and DHA balance is further demonstrated in an analysis of the effect of LCPUFA levels on ROP-risk in Sweden, concluding that higher levels of DHA were only associated with a reduced risk of ROP if the levels of AA were sufficiently high [37].

Premature birth itself may also be linked to inflammatory signaling, as illustrated by the actions of prostaglandins to precipitate preterm birth in sheep [38]. In humans, mothers whose blood content of AA + DHA was <1.6% of total blood fatty acids had 10-fold higher risk of premature birth in comparison to mothers with LCPUFA levels >1.8% [39]. A recent study showed that the DHA and AA status of cord blood correlates with levels of the inflammatory markers CRP and IL-6 of prematurely born infants [40], while inflammation in the fetal and early post-natal periods are linked with increased morbidity in later life [41,42]. 

## 3. LCPUFA Accretion into Fetal Tissues 

### 3.1. In Utero LCPUFA Accretion

The final trimester is associated with rapid growth of tissues, including adipose tissue, skeletal muscle, and brain tissue [43]. Between 31 weeks of gestation and term, the brain increases in size from ~150 mL to ~400 mL [44], while brain weight increases by four- to five-fold [45]. This tissue growth is matched by an increased transfer of fatty acids from mother to fetus via the placenta to provide both energy and structural substrate for building new tissue. Furthermore, the placenta serves to selectively control the transfer of both AA and DHA from mother to infant. In a process termed biomagnification, the placenta transfers LCPUFA to the fetus even during circumstances of maternal LCPUFA depletion, resulting in higher LCPUFA contents of fetal blood and tissues than those of the mother [43].

AA transfer is high throughout pregnancy, with the biomagnification phenomenon maintaining fetal blood AA at levels twice those of the mother; at the beginning of the third trimester, the AA:DHA ratio is ~5.1 [46]. The rate of LCPUFA transfer across the placenta increases significantly in the final trimester, increasing from an average of 6.1 mg of AA and 2.3 mg of DHA per day in the 25th week of gestation to 95 mg AA and 42 mg of DHA per day in the final trimester [47]. The accelerated transfer of DHA in the third trimester results in fetal cord plasma DHA levels exceeding those of the mother at around 33 weeks [46]. At term, the AA:DHA ratio as measured in fetal cord blood is ~2.5:1 [46]. Changes in brain LCPUFA composition correspond with these blood LCPUFA changes, and between the gestational ages of 8 to 40 weeks, the relative brain content of AA decreases (~11% to 8.6%) and DHA content increases (3.2% to 8.4%) in infants born to women eating a traditional diet rich in DHA and without excessive LA [48]. 

### 3.2. Post-Natal LCPUFA Accretion

Accretion of LCPUFA continues in the postnatal period and throughout childhood up until the age of 18, but at a decreasing rate [49]. Maternal breastmilk is a rich source of LCPUFA in the postnatal period and contains ~0.6% AA and ~0.3% DHA; however, these concentrations show considerable variation with maternal diet [50] and genetic polymorphisms [1]. Over the first six months of life, breast fed infants accumulate DHA at ~10 mg/day [51].

### 3.3. LCPUFA Accretion into Adipose

While much of the LCPUFA transferred to the fetus in the final trimester contributes to brain growth and neurological development, by term, 90% of all maternally derived energy, including LCPUFA, is deposited into adipose tissue to result in seven-fold more DHA stored in adipose than in brain tissue [52], with the clinical sequela of this being that this DHA enrichment of adipose tissue is not afforded to prematurely born infants. 

It is estimated that the deposition of N3 and N6 fatty acids into adipose tissue outweighs that of brain and other neural tissues by ~46-fold, consuming 78% and 70% of all N6 and N3 PUFA transferred from mother to infant [53]. While total N3 and N6 content in adipose increases, their percentage relative to other fatty acids decreases; AA and DHA similarly decrease from ~4% to ~0.4% between 19 to 38 weeks of gestation [48]. Indeed, the storage of both energy and LCPUFA is essential for ongoing tissue, brain, and neurological development in the post-natal period, in which a steady supply of nutrition from maternal breastmilk is not guaranteed [54].

The importance of LCPUFA stored in adipose tissue is demonstrated by Cunnane et al., who compared the LCPUFA tissue content of infants fed either breastmilk or LCPUFA-deficient formula in the postnatal period [51]. Formula-fed infants increased the LCPUFA content of their brains, but at the expense of their adipose tissue DHA stores, which were depleted to unmeasurable levels. On the other hand, the breastfed infants increased both brain and adipose contents of DHA [51]. These findings demonstrate that, while adipose stores of LCPUFA can be mobilized to supply the brain, they are likely to be inadequate to fully support neurodevelopment and must be augmented by ongoing nutritional LCPUFA support postnatally. 

In the absence of normal intrauterine LCPUFA supply, the deficits incurred by premature birth can be severe; an infant born at 35 weeks, despite being born at an appropriate weight for gestational age (AGA), will have half the LCPUFA content stored in their tissues of an AGA infant born at term [55]. This ‘gap’ increases exponentially with the increase of prematurity [46]; an infant born at 25 weeks may weigh 1300 g and have adipose stores 10% of those of a term infant [53]. 

In summary, the final weeks of gestation are a key period for accretion of these fatty acids, with LCPUFA stores and adipose tissue mass effectively doubling. Premature birth disadvantages infants two-fold, by limiting DHA accretion directly into neural tissues as would happen in utero and by hindering the accumulation of adipose stores that the infant must rely on to continue optimal post-natal brain development.

## 4. What Is the Evidence Linking Maternal and Neonatal PUFA Intake with Neurodevelopmental Outcomes?

There is a heterogeneous population of studies assessing the effects of LCPUFA supplementation and neurodevelopmental outcomes of prematurely born infants. Supplementary Table S1 aims to describe and summarize these studies with respect to the size, age, and weight of the preterm population studied, the concentrations of AA and DHA provided, the presence and concentration of other LCPUFAs such as LA and ALA, and the duration of supplementation. The majority of studies measure neurodevelopment using the Bayley Scales of Infant Development (BSID); however, additional tools such as the Ages and Stages Questionnaire are used, while complementary studies of brain volume or visual acuity are also employed. 

### 4.1. Randomized Controlled Trials

Two randomized controlled trials (RCTs) have studied neurodevelopment with regard to varying AA:DHA or N6:N3 ratios in premature infants. Alshweki et al. [8] studied the effects of formula feed containing a variable N6:N3 ratio upon neurodevelopment in a population of 60 premature (<32 weeks) or low birth weight (<1500 g) infants. Formula N6:N3 ratios of 2:1 and 1:1 were produced by using a fixed dose of DHA (0.3%) and varying AA content (0.3%/0.6%) and did not contain LCPUFA other than AA/DHA. These formulas were provided for 12 months and compared against age-matched controls fed exclusively human milk. At 12 months, the infants fed an AA:DHA ratio of 2:1 had significantly greater blood levels of AA in comparison to the 1:1 group and the breastfed group. Infants fed the 2:1 formula scored similarly to breastfed infants and were superior to infants fed the 1:1 formula on neurodevelopmental tests at 24 months of age [8]. These results suggest that an AA:DHA ratio of 2:1 produces neurodevelopmental outcomes more similar to breastfed infants in comparison to a ratio of 1:1 and that AA concentrations of 0.3% may be suboptimal for neurodevelopment. 

The second trial to vary the AA:DHA ratio was the multicentre DINO trial [10]. Preterm infants (<33 weeks) born in five different hospitals in Australia were randomized to either a diet providing ‘high’’ amounts of DHA as 1% of fatty acids or a control diet containing a ‘standard’ 0.3% DHA concentration representing typical feed. Breastmilk was encouraged and supplemented to achieve a ‘high’ DHA concentration, or supplemented formula feed was provided. Both breastmilk and formula were reported to contain AA as 0.6% of fatty acids. The DINO study did not report the percentages or amounts of LA and ALA included in the feeds. This study employed a DHA concentration of 1% to mimic the intrauterine DHA accumulation rate, and to simulate a pregnancy continued until term, infants were fed soon after premature birth until term corrected age (CA). Measurement of neurodevelopment at 18 months found no difference between the high- or standard-DHA groups with respect to the Motor Development Index (MDI) component of the BSID. However, the study found that fewer infants in the ‘high’ group scored below 70 points, indicating that ‘high’ DHA supplementation may have aided the infants at greatest risk of neurodevelopmental impairment, such as those born at greater immaturity or at the lowest birth weights [10]. Similarly, MDI scores were greater for low-birth weight infants weighing <1250 g, but this result was not significant after adjustment [10]. Follow up at 26 months CA found no effect of DHA supplementation on visual, language, or behavioral development [11], and no benefit to IQ was measured at 7 years of age [9].

Studies in Norway assessed the effects of LCPUFA-supplemented breastmilk for a median duration of 9 weeks to 141 premature infants weighing <1500 g and born at a mean GA of 28 weeks [56]. Breastmilk was supplemented to achieve AA and DHA concentrations of 1% and 1.5% (of total fatty acids), respectively. Plasma DHA increased in the intervention group and decreased in the control group, suggesting DHA utilization but with inadequate replacement in the control group. Plasma AA decreased in both intervention and control groups, but more so in the control group, indicating depletion of AA with inadequate dietary replacement. Similar consumption of LCPUFA stores have been found in the tissues of infants fed LCPUFA-depleted breastmilk [51]. Plasma DHA at discharge from the hospital was associated with the Bayley MDI and with measures of ‘sustained attention’ [57]. Neurodevelopmental tests at 18 months found no difference in overall test scores between groups; however, the LCPUFA-supplemented group scored significantly higher on a sub-test assessing problem-solving ability. At 20 months of age, assessment during free-play found that infants in the LCPUFA group had greater ‘summary attention ratings’ versus controls [57]. Later assessments at 26 months found no differences between MDI scores, while at 8 years of age no anatomical changes on MRI [7,58] and no differences in IQ [59] were evident. 

In addition to Alshweki et al., Clandinin et al. [60], and O’Connor et al. [61] studied the effects of LCPUFA-supplemented feeds given for 12 months. Clandinin et al. [60] studied 245 preterm infants born before 36 weeks GA weighing <1500 g. Two interventional groups of infants were fed formula containing 0.3% DHA and 0.6% AA to simulate the concentrations found in breastmilk, while the feeds also contained LA and ALA within ranges of 17–19% and 1.5–2.5%, respectively, to achieve a N6:N3 ratio of ~9:1. Comparisons were made against preterm babies ingesting formula deficient in both AA and DHA, and they were made against healthy term-born breastfed infants. Neurodevelopment was measured at 18 months using the Bayley MDI and Psychomotor Development Index (PDI) components. Preterm infants fed DHA-containing feeds achieved better MDI and PDI scores at 18 months than premature infants fed a control diet but did not score as high as the breastfed term infants. 

O’Connor et al. [61] studied the effects of formula containing 0.15–0.27% DHA and ~0.4% AA provided for 12 months. These formulas included 16–20% LA and ~2.5% ALA to achieve a ~8:1 N6:N3 ratio. A large population of 470 infants, born <33 weeks and weighing 750–1805 g, was recruited from multiple centers across two countries. At 12 months, there were no differences in BSID scores between groups. Subgroup analyses found improved MDI scores amongst infants supplemented with DHA at 12 months who had been born at a very low birth weight (<1250 g).

A further two studies evaluated the effects of 6 months of LCPUFA supplementation in small groups of infants. Van Wezel-Meijler et al. [62] randomized 42 pre-terms (<34 wks GA) with low birth weights (<1750 g) to either LCPUFA-supplemented diets containing DHA (0.34%) and AA (0.68%) or to formula without LCPUFA, fed until 6 months CA. Neither feed contained any ALA or LA. MRI studies performed at 3 and 12 months found no differences in myelination, and no differences between BSID scores were found at 3, 6, 9, and 12 months. The percentages of AA and DHA in the intervention feed represent those of breastmilk, and are comparable to those used in Clandinin et al., the control group in the DINO trial, and the 2:1 group of Alshweki et al. 

On the other hand, Fang et al. [63] used standard infant formula with an LA:ALA ratio of 10:1, compared against the same formula with concentrations of 0.05% DHA and 0.10% AA. The population studied was of larger and more mature preterm infants, born between 30–37 weeks and weighing >2000 g, and the intervention lasted for 6 months. Although this study used lower DHA and AA concentrations relative to other groups and to maternal breastmilk, significantly higher MDI and PDI scores were found in the LCPUFA group at both 6- and 12-months CA. 

A collection of studies led by Fewtrell et al. measured neurodevelopmental outcomes after different durations of feeding with formulae of multiple LCPUFA compositions. In 2002, Fewtrell et al. [64] published the neurodevelopmental outcomes at 9 and 18 months of infants who had been born at a mean GA of 30 weeks weighing 1300–1400 g and fed supplemented formula from 10 days until discharge (for a mean duration of 37 days). DHA was provided at 0.17%, AA at 0.31%, LA at 12%, and ALA at 0.6% of fatty acids, and they were compared against formula deficient in AA and DHA but containing LA and ALA at 10% and 0.7% of fatty acids, respectively. In addition to the BSID second edition (BSID-II), the group used Knobloch, Passamanick, and Sherrard’s Developmental Screening Inventory (KPS). No differences in neurodevelopment measures were found between groups at either 9 or 18 months; however, there was a significant interaction between gestational age at birth, diet, and MDI at 18 months of age. Infants born at greater prematurity and fed LCPUFA formula had non-significantly better scores on both MDI and PDI at 18 months than infants fed LCPUFA depleted formula, but this relationship did not exist for infants born after 30 weeks [64]. 

Subsequently, the Fewtrell group (2004) studied a similar cohort of infants born at a mean GA of 31 weeks and mean birthweight ~1500 g, extending the duration of feed until 9 months CA [65]. A different formula fatty acid composition was employed, comprising 0.5% DHA, 0.04% AA, 1.5% ALA, and 12.3% LA. Control formula was AA- and DHA-deficient, but it contained ALA at 1.6% and LA at 11.5%. Thus, despite an increase in DHA and ALA, reduced AA content, and extended intervention duration, no differences in neurocognitive outcomes measured by the BSID-II were found at 9 or 18 months [65] or at follow up at 9 years [66]. 

Two older studies evaluated the sole provision of the N3 LCPUFA DHA and EPA. Carlson et al. (1996) [67] studied very low birthweight infants (<1390 g) born at a mean gestation of 29 weeks who were provided feed containing 0.2% DHA from enrollment at days 2–5 of life until 2 months post term. After this time, infants continued to be fed AA- and DHA-deficient formula containing 34% LA. Both intervention and control feeds contained LA and ALA at a ratio of 6.5–7:1. No difference was found between the intervention group and infants fed a standard EPA- and DHA-deficient feed with regards to cognitive development, tested by the 52-week (12-month) Fagan test, administered at 92-wk PMA. 

In a similar population of preterm infants <33 weeks GA (mean 29 weeks) and weighing <1400 g (mean 1103 g), Werkman et al. [68] provided a commercial formula with or without the addition of marine oil to achieve concentrations of 0.2% DHA, provided until 9 months CA. Both control and intervention feeds contained 2.4–4.8% ALA and 21% LA. Intelligence was measured via the Fagan test at 6.5, 9, and 12 months. These studies speculate that the DHA-supplemented groups displayed some signs of superior information processing capabilities [67,68]. 

These studies insofar have excluded infants with comorbidities associated with premature birth. The UK based Dolphin [69] trial identified infants born prematurely (<31 weeks) and at risk for suboptimal neurodevelopment due to concomitant comorbidities such as IVH, WMI, hypoxic-ischaemic encephalopathy, or neuroimaging abnormalities. A dietary supplement containing DHA, EPA, AA, choline, and uridine-5-monophosphate, the latter two being critical components of phospholipids, was given for two years. DHA was provided at 1% of total daily intake of fatty acids, while no other supplemental LCPUFA were described by the authors. A composite score including the BSID-III was used to measure neurocognitive changes at 24 months but found no significant group differences. Limitations included poor compliance with the intervention; only 66% of infants in the intervention group completed the full course of supplementation. 

Finally, the Preemie Tots [70] trial sought to assess the effects of LCPUFA upon parentally-reported symptoms and behaviors of 31 infants at risk of developing Autistic Spectrum Disorder, born prematurely but aged between 18–38 months at the time of study. Parents evaluated their children using the BITSEA ASD scale after 90 days of supplementation of N3, N6, and N9 LCPUFA, finding improvements in comparison to infants fed canola (rapeseed) oil. 

### 4.2. Reviews and Systematic Reviews 

A 2016 Cochrane systematic review found no statistically significant relationship between LCPUFA supplementation and neurodevelopment measured by BSID and indicated that the overall quality of evidence available was low [6]. Alternative meta-analyses by Wang et al. and Shulkin et al. both found benefits to MDI scores in preterm infants supplemented with LCPUFA [71,72]. Lapillone and Moltu agreed that supplementation with larger doses of DHA than standard is associated with greater neurological outcomes between 18 months and 2 years [73]. Most recently, Klevebro et al. discussed the seven trials analyzed in the Cochrane review with two additional trials that compared formulae with different ratios of LCPUFA [74]. These reviews and meta-analyses shared similar criticisms of the available evidence, namely the lack of consistency between studies regarding methodology, timing of supplementation, dosage of supplemental LCPUFAs, source of LCPUFAs and the fatty acid composition of the control formula.

## 5. Can the Evidence Inform Future LCPUFA Supplementation Strategies?

Although the studies above share few consistent concentrations of AA and DHA, the majority employ concentrations resembling the recommendations of the European Academy of Paediatrics and Child Health Foundation (2020) for formula to contain at least 0.3% but preferably 0.5% DHA, while AA should be at least equal to DHA content [75]. 

### 5.1. Is Supplemental LCPUFA Better than None at All? 

Alshweki et al. showed that a formula containing 0.3% DHA and 0.6% AA (AA:DHA ratio 2:1) is at least better than LCPUFA-deficient formula in the absence of other LCPUFA (LA and/or ALA), demonstrating that these ratios were associated with neurodevelopmental scores most closely resembling those of breast-fed infants [8]. Clandinin et al. provided the same concentrations of AA and DHA as Alshweki et al., with an LA:ALA ratio of 9:1, for 12 months to achieve better neurodevelopmental outcomes than infants fed no LCPUFA [60]. These positive results are not unanimous, however; van Wezel-Meijler et al. fed 0.34% DHA and 0.68% AA with no additional LCPUFA for 6 months but found no differences in physical or cognitive measures of neurodevelopment [62]. The obvious caveat to be acknowledged is that preterm infants are exposed to a number of risk factors for neurodevelopmental impairment beyond differences in fatty acid accumulation.

### 5.2. Would Higher Doses of Supplemental LCPUFA Provide Greater Benefits to Neurodevelopment?

If 0.3% DHA and 0.6% AA appears to benefit infants, is there benefit to providing higher concentrations to mimic the physiological LCPUFA transfer rate of the placenta more closely? 

**Figure 1:**
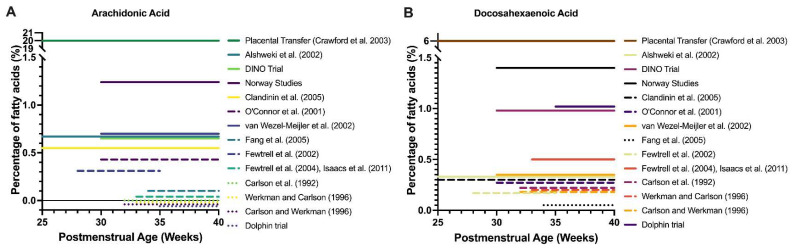
Clinical trials of LCPUFA supplementation in premature infants. Timing and dose of AA (**A**) and DHA (**B**) supplementation as percentage of fatty acids by trial and compared to estimated placental transfer. Note the y-axis breaks, which show that DHA and AA supplementation were at least 4–10 times below levels of placental transfer in the third trimester, regardless of the trial. For AA, all lines below 0% represent 0% of fatty acids as AA but have been separated for display purposes.

The concentrations of DHA and AA in standard feed are lower than the amounts of LCPUFA provided by the placenta in the final trimester, estimated at >6% of fatty acids for DHA and 14–20% of fatty acids for AA. The quantities of LCPUFA provided by standard formula feed is therefore around 10- to 50-fold less than the physiological expectation during the post-natal period after preterm birth [76]. Current feed with low LCPUFA content fails to meet the in-utero rates of DHA accretion [43,55] and is recognized to contribute to large LCPUFA deficits even in term infants [51], hence it is expected to worsen the deficit incurred by preterm birth. Figure 1 demonstrates the DHA and AA content of feeds employed in studies compared to physiological DHA and AA provision by the placenta. 

Thus, there may be benefits to providing greater concentrations of LCPUFA than those given at present. DHA given at 1%, mimicking the ‘high end’ of breastmilk and still less than the transfer rate possible by the placenta, increases the brain DHA content of term neonatal baboons by 40% in comparison to 0% DHA feed [77]. Human evidence, however, is limited. There are no human infant studies mimicking placental LCPUFA transfer rates and few that provide more than the minimum percentage concentrations recommended by the European Academy of Paediatrics and Child Health Foundation. The DINO trial provided 1% DHA and 0.6% AA to infants during the period between premature birth and their expected term date but did not find overall group benefit [10]. Fewtrell et al. provided 0.5% DHA but found no cognitive benefit, although this result is confounded by a low concentration of AA (0.04%) [65]. In term infants, the DIAMOND study found no benefit with provision of DHA above percentages of 0.32%, while DHA at 0.64% resulted in imbalances of the AA:DHA ratio in erythrocytes [78,79]. Whether provision of even greater DHA and AA concentrations (~6%) that mimic the in-utero transfer rate of the placenta is feasible and have positive outcomes is as of yet unknown. 

### 5.3. Which Preterm Infant Population May Benefit Most from LCPUFA Supplementation?

An interesting relationship between the degree of prematurity and benefit from supplementation is apparent, whereby infants born at greater immaturity may benefit more from LCPUFA supplementation. The DINO trial, providing 1% DHA and 0.6% AA, found improved neurodevelopment in the most premature or lowest birthweight infants but not in whole group analyses, including preterm infants of greater maturity [10]. O’Connor et al. found no neurodevelopmental differences amongst their whole cohort of 470 infants, but significant improvements to MDI score were found amongst infants born at a low birthweight [61]. Similarly, Fewtrell et al. found an effect of LCPUFA supplemented formula upon MDI scores of infants born before, but not after 30 weeks GA, using formula comprising 0.17% DHA and 0.31% AA with an LA:ALA ratio of 20:1 [64]. These extremely premature or low birthweight infants have the greatest LCPUFA deficits relative to healthy infants born at term, which may explain the benefits seen in this population despite variation of the concentrations of AA, DHA, and other LCPUFAs provided between studies. 

### 5.4. Tailoring Supplemental DHA and AA to Mimic Dynamic Physiological Placental Supply and Developmental Demand

A further aspect to consider is the changing rate and ratio of placental LCPUFA transfer across gestation. Martinez et al. showed that in early gestation, transfer of AA is predominant, while DHA transfer is delayed until the final weeks of gestation [49,80]. This differential transfer of AA and DHA with time results in dynamic N6:N3 ratios, of 5:1 at the beginning of the third trimester and ~2:1 by term [46]. The rate of transfer of DHA only exceeds that of AA at 33 weeks’ gestation [46]. After birth, DHA accretion into brain continues to peak between 2 and 3 years of age, while brain AA content remains relatively unchanged [49]. 

Current feeding regimes for preterm infants provide static concentrations of supplemental AA and DHA, but in pursuit of a greater depth of physiological mimicry, we may wish to vary formula AA and DHA concentrations with respect to infant CA to better simulate the dynamic functions of the placenta and demands of the infant. In this regard, it may be that prematurely born infants require several formulae during their postnatal period; an infant born at 25 weeks may require more AA than DHA, whilst an infant born after 33 weeks may require more DHA than AA. Formula provided after the premature infant’s ‘term’ date (corrected age) should resemble the LCPUFA composition of breastmilk for ongoing postnatal nutrition. Looking onwards, the LCPUFA composition of weaning foods and ongoing diet may be considered to be modifiable factors affecting LCPUFA assimilation and accumulation in childhood and later life. 

### 5.5. Organ-Specific Effects of LCPUFA 

It is apparent that LCPUFA availability has differential effects upon the development of specific organs. Although postnatal supplementation of DHA and AA seem to be beneficial for neurodevelopment and retinal development in preterm infants [16,32], recent studies have indicated a potentially opposing effect in pulmonary development, with no benefit on rates of—and a potentially increased risk for—bronchopulmonary dysplasia (BPD) in infants supplemented with LCPUFA [35,36,40]. This is despite pre-clinical evidence and observational studies showing that lower postnatal levels of DHA are associated with increased risk of chronic lung disease [34,81]. Reasons for this are unknown, but supplementation with DHA might lead to alterations in the levels of other LCPUFAs important for pulmonary development or an imbalance in fatty acid metabolites. More widely, supplementation with single specific fatty acids might have different effects on different organs. We must refine our approach to LCPUFA supplementation to holistically optimize the development of all organ systems. 

### 5.6. Challenges for Appropriate LCPUFA Supplementation

Premature birth brings many uncertainties and unexpected physiological challenges for the newborn, for which it is unprepared. Examples include exposure to air and increased oxygen tensions, infections, and varying types of brain and peripheral organ injury. LCPUFAs may be protective via their metabolism to eicosanoids and SPMs yet are also susceptible to peroxidation, which may propagate cellular damage [76,82,83]. We may consider whether more or less of individual LCPUFA or modifications of the N3:N6 ratio may help or hinder both normal development and resilience against challenges associated with prematurity. 

Regardless of the amount of supplemental DHA provided, we must also consider the effectiveness of the route chosen to provide DHA to prematurely born infants. Administration of high amounts of fatty acids such as DHA via the immaturely developed premature infant digestive system may be ineffective or problematic due to altered absorption. Additionally, intolerance of oral feeding and gastrointestinal complications such as spontaneous intestinal perforation and necrotizing enterocolitis are common with increasing prematurity. Parenteral lipid supplementation is therefore common in these infants but also does not fully mimic or replace normal fatty acid metabolism.

Overall, supplementation of LCPUFA is greatly nuanced and has the potential to both benefit and harm the premature infant. Pragmatically, future research must also consider that measurement of fatty acid levels in blood might not correlate with the levels in specific tissues. It is evident that a greater understanding of changes in LCPUFA transfer rates with gestational maturity and the effects of LCPUFA availability upon specific tissue development is needed to optimize the growth and development of prematurely born infants. 

## 6. The Wider Contexts of LCPUFA Supplementation

### Dietary LA Affects Infant AA and DHA Availability 

In addition to large differences in AA and DHA concentrations across the trials to date, there is large variation between the concentrations of LA and ALA provided in both control and intervention formulae. Historical formulae have included LA and ALA with an assumption of adequate endogenous conversion to AA and DHA, but in reality, this conversion is inadequate to meet infant demands. For instance, Cunnane et al. showed that infants fed DHA-deficient formula depleted their adipose stores to unmeasurable levels and had less brain DHA accumulation than infants fed DHA-repleted formula [51]. Other autopsy studies have shown reduced total DHA content of brains of infants fed formula that was deficient in DHA, despite containing LA and ALA [18]. 

Similarly, Bockmann et al. showed that feeding LA-rich feed to preterm infants alters the adipose LCPUFA composition to become dominated by LA and depleted of AA and DHA. These results show that, while dietary ALA and LA are inadequate to supply infant needs of DHA and AA, excessive LA is stored and persists in fetal adipose tissue [84]. Furthermore, these preterm infants had reduced AA and DHA bound to phosphatidylcholine in their blood, suggesting a reduction in total availability of AA and DHA to developing tissues, such as brain tissues. 

Instead, excess LA in infant diet and infant tissues has the potential to alter the LCPUFA composition of multiple tissues, as well as the overall N6:N3 ratio. This ratio is of significance due to metabolic competition at the level of the desaturase enzymes, whereby excesses of one series of LCPUFA may out-compete others for metabolism into downstream metabolites, contributing to a relative deficiency of these other LCPUFA despite adequate oral intake. It is known that dietary PUFA composition can alter the N6:N3 balance of tissues in both animals and humans. Hsieh et al. demonstrated competition between AA and DHA in infant baboons who were given feed containing varying N6:N3 ratios by providing a fixed percentage of AA and ‘standard’, ‘medium’, or ‘high’ percentages of DHA. The baboons with the highest DHA intake demonstrated a reciprocal decrease in brain AA content, representing competition between N3 and N6 fatty acids for placement within neuronal membranes [77]. Infant piglets fed formulae containing comparable ALA (N3) but with differences in LA (N6) content demonstrated changes in tissue LCPUFA composition to resemble those of their diets; piglets fed N6-rich formula had greater N6 content of liver, blood, retina, and brain, but they also showed relative depletion of N3 LCPUFA in comparison to piglets fed sow milk with a more equal N3:N6 ratio [85]. In a further study of piglets by the same group, increased duration of LA-rich (30%) feeding associated with progressive decreases in retinal and brain DHA and reciprocal increases in N6 LCPUFA [86]. 

Similarly, findings have been seen in human infants born >35 weeks GA provided total parenteral nutrition (TPN) high in LA for a maximum of 12 days after birth. In these infants, hepatic levels of LA increased three-fold, while levels of DHA halved, with a consequent dramatic reduction in the N3:N6 ratio [87]. While this study found no change in brain DHA content at autopsy during its <11-day duration, the effects of more persistent dietary manipulation of the N3:N6 ratio upon human neurodevelopment is unknown. 

The DIAMOND [78,79] trial is the only study to provide a varying ratio of N3:N6 and measure neurodevelopment. Term infants were fed formula containing a fixed dose of AA (0.64%) with varying percentages of DHA (0.32% DHA, 0.64% DHA, or 0.96% DHA) to achieve N6:N3 ratios of 2:1, 1:1, and 0.7:1. These DHA-supplemented groups were compared against a control cohort fed formula containing 0% DHA and 0% AA. All formulas contained similar amounts of LA (16.9–17.5% fatty acids) and ALA (1.61–1.68% fatty acids). 

Infants in the DIAMOND trial that were fed formula containing DHA had improved visual acuity at 3 months and superior Bayley Scale of Infant Development (BSID-II) Motor Development Index (MDI) scores, emotional regulation, and language abilities at 18 months in comparison to infants that were fed the formula containing 0% DHA [78,79]. Furthermore, improved language and cognitive performance were seen between ages 3–5 years [88], while at 9 years, MRI measures of connectivity, brain volume, and neurochemical levels were greater in children fed DHA-containing formula [89].

The DIAMOND studies generally found no benefit to greater concentrations of DHA in feed; the majority of benefits were found at a ratio of 2:1 or 1:1, with no significant benefit found at lower N3:N6 ratios or higher DHA concentrations. At greater intakes of 0.96% DHA, erythrocyte DHA increased with a reciprocal decrease in AA to reach a level below those of infants fed AA-deplete formula, confirming the findings of other studies demonstrating interaction and displacement between series of LCPUFA [78]. 

Furthermore, there appear to be more complex synergistic effects between AA and DHA. The Mega Donna Mega trial analyzed DHA and AA serum concentrations with regard to risk of ROP development in premature infants and found that higher serum DHA only conferred reduced ROP risk when AA was above a threshold concentration; the same DHA concentration did not reduce ROP risk in infants with low serum AA [21].

The cumulative storage of and interaction between dietary AA, DHA, and their parent LCPUFAs likely represent an important confounding factor that may affect our interpretation of all LCPUFA-supplementation studies that report neurocognitive outcomes at timepoints in later childhood. After feeding until 9 months, Fewtrell et al. found no neurocognitive benefits a [65] or 18 months or at 9 years [65]. The studies from Norway supplemented breastmilk with AA and DHA for 9 weeks but found no overall differences in neurodevelopment at 18 months, 26 months, or at 8 years [7,59]. Similarly, the DINO study, providing supplemented feeds until term corrected age, found no benefit at 7 years of age [9]. Due to the role of adipose tissue to sequester LCPUFA and the ongoing competition between N3 and N6 LCPUFA families for metabolism and distribution into tissues or membranes, even the prolonged supplementation periods demonstrated in the studies above are relatively short in comparison to the interim between the end of the supplementation period and later neurocognitive measurements. This intervening period is unaccounted for by these studies, with infants returning to formula deficient of AA/DHA or of variable ALA/LA content before weaning onto a diet of uncharacterized LCPUFA composition. The LCPUFA composition of post-intervention formula and weaning foods may partly explain the lack of effect of LCPUFA-supplementation during the perinatal period upon neurocognitive outcomes measured years after birth. These foods should be considered as important factors to be controlled, or recognized as being confounders, when designing future studies that will assess neurocognitive performance throughout childhood development.

In summary, there is evidence for an interaction between AA, DHA, and other essential LCPUFA in animal models and both preterm and term humans. The DIAMOND study is the only evaluation of the effects of the N3:N6 balance and found neurodevelopmental benefits across multiple time points in childhood but is specific to infants born at term [78,79]. 

## 7. The Wider Factors Affecting LCPUFA Status 

### 7.1. Maternal LCPUFA Status

The maternal fatty acids that supply the infant in utero and in breastmilk are derived from the maternal diet and stores in adipose tissue [90]; the quality of fatty acids consumed and stored by a prospective or pregnant mother is a biologically plausible and modifiable factor affecting infant developmental outcomes after premature birth. The Western diet has become accustomed to an increased consumption of N6 PUFA-rich foods, such as vegetable oils [91], a dietary change that is reflected in the fatty acid composition of maternal tissues. 

In the 1980s, Clandinin et al. remarked that the N6 content of fetal adipose tissue was higher than previous studies, attributing this to changing maternal dietary habits to include more vegetable oils [53], intakes of which now far exceed the nutritional requirement for N6 LCPUFA of ~1% fatty acids [92]. Indeed, differences in fatty acid composition of breastmilk and adipose tissue have been seen between traditional and modern populations [50,90,93]. 

### 7.2. Genetic Polymorphisms

Aside from diet, genetic polymorphisms of the FADS gene affecting expression of the fatty acid desaturase enzymes that convert LCPUFA precursors to AA and DHA may affect maternal LCPUFA synthesis and thus availability of LCPUFA to the infant. FADS gene polymorphisms may reduce the maternal ability to provide LCPUFA in breastmilk [1,94] and correlate with reduced infant IQ [95]. In case of genetic variants corresponding to reduced LCPUFA conversion, maternal intake of preformed AA and DHA, or specific supplementation of formula, may be crucial to provide adequate AA and DHA to the infant both in-utero and in the postnatal period [96]. 

### 7.3. Socioeconomic Status

Dietary LA also increases with decreasing socioeconomic status [92]. One of the two cohorts of the DIAMOND study represented families of lower socioeconomic status; infants born into these families demonstrated greater levels of erythrocyte AA than their peers born into families living in wealthier areas [78], illustrating a potential relationship between socioeconomic status, maternal and infant intake of foods rich in N6 PUFA, and early life development [74]. It is of interest that neurodevelopmental outcomes of infants whose mothers supplemented with DHA during pregnancy are more pronounced in families of lower education or of ‘poorer’ home environments [97,98]. It may be that families of lower SES may derive the most benefit from strategies to decrease dietary N6 intake and/or supplement with N3 LCPUFA. 

Maternal LCPUFA status may also affect the trajectory of infant development with regard to gestation length and in-utero development. A 2018 paper found that low maternal blood EPA and DHA concentrations increased risk of preterm birth by 10-fold [39], and a recent Cochrane review found that maternal N3 supplementation was associated with a lower risk of both preterm (<37 weeks) and early preterm birth (<34 weeks), infant perinatal death, and low birthweight, suggesting that optimal maternal LCPUFA status may prevent preterm birth and influence the risk of many neonatal comorbidities [99]. 

On the other hand, results from large cohort studies that assessed the overall dietary LCPUFA intake during pregnancy have suggested that increases in the N6:N3 ratio are associated with developmental delay. Kim et al. estimated the N3 and N6 PUFA intakes of 960 mothers during pregnancy, and of their infants via breast milk and weaning feeds in the postnatal period, to assess the effects of N3 and N6 intakes upon neurodevelopment at 6 months as measured by BSID-II; greater LA:ALA and N6:N3 ratios were both associated with lower MDI and PDI scores in infants, while multiple logistic regression analysis showed that infants of mothers with the highest LA:ALA ratios had >2 times the risk of neurodevelopmental delay of infants whose mothers had the lowest LA:ALA ratios [100]. Similar findings of a negative correlation between maternal N6:N3 ratio and language ability in infants born at term and not breastfed were found by Bernard et al. [101].

After birth, increased maternal N6 LCPUFA status may directly affect infant tissue LCPUFA composition via the breastmilk, while weaning onto a N6-rich modern diet may contribute to ongoing competition between N6 and N3 fatty acids. The fatty acid composition of maternal milk and the wider food environment are unaccounted factors that may influence the results of trials assessing the long-term effects of DHA in the post-natal period. 

In summary, it is evident that the maternal dietary intake and storage of LCPUFA, in combination with genetic factors, can affect the availability of these LCPUFA to developing infants and affect the consequent fatty acid composition of infant tissues. Furthermore, a suboptimal maternal LCPUFA status may increase the risk of preterm birth, while a positive LCPUFA status may improve infant growth and thus reduce the risk of neonatal morbidity. Whether maternal LCPUFA status affects neurodevelopment in preterm infants is yet unknown and is largely unstudied, thus the authors welcome future studies incorporating data of maternal LCPUFA status into calculations of absolute and relative LCPUFA availability to the developing infant. 

## 8. Conclusions

LCPUFA transfer from mother to fetus occurs via the placenta throughout pregnancy, with particularly high rates of DHA transfer during the final trimester. LCPUFA accrete into brain tissue and are essential for neurodevelopment; however, the bulk of LCPUFA are stored in adipose tissue to provide vital endogenous LCPUFA stores in the postnatal period. Infants born before term do not receive this placental transfer of LCPUFA and hence can be severely LCPUFA-deficient in both brain and adipose tissue in comparison to term-born infants. 

The evidence supporting post-natal LCPUFA supplementation for prematurely born infants is weakened by heterogeneity within the populations studied, durations of feeding and the concentrations of AA, DHA, LA, and ALA provided. While firm conclusions cannot be drawn, it is apparent that providing DHA at 0.3% and AA at 0.6% provides greater benefit to the neurodevelopment of prematurely born infants than feeding LCPUFA-deficient formula. 

Future studies should consider the absolute and relative availability of AA and DHA and the timing of these fatty acids with respect to normal placental physiology. Furthermore, the availability of other LCPUFA such as LA and ALA within infant formula should be seen as factors interacting with the relative availability of AA and DHA within the infant. More widely, the role of maternal LCPUFA status is poorly understood, but may directly affect the risk of premature birth and infant neurodevelopment. 

## Data Availability

Not applicable.

## Supplementary Materials

The following are available online at https://www.mdpi.com/article/10.3390/ijms23020700/s1.
