# Supplementary material for: Maternal and Neonatal Polyunsaturated Fatty Acid Intake and Risk of Neurodevelopmental Impairment in Premature Infants
Source: Int J Mol Sci. 2022 Jan 9;23(2):700. doi: 10.3390/ijms23020700 (PMC8775484; doi:10.3390/ijms23020700)
Supplement: Supplementary file 1 [file ijms-23-00700-s001.zip › ijms-1525694-supplementary.pdf]

International Journal of
Molecular Sciences

MDPI

Table S1. Summary table of studies relating LCPUFA supplementation and neurodevelopmental outcomes of prematurely born infants with respect to the size, age and weight of the preterm population studied, the concentrations of AA and DHA provided, the presence and concentration of other LCPUFAs such as LA and ALA, and the duration of supplementation.

Abbreviations: BW: Body weight, GA: Gestational Age, CA: Corrected Age, DHA: Docosahexanoic Acid, AA: Arachidonic acid, ALA: Alpha-linoleic acid, LA: Linolenic Acid, GLA: Gamma-linolenic acid, EPA: Eicosapentaenoic acid, LCPUFA: Long chain polyunsaturated fatty acid, UMP: Uridine-5-monophosphate, BSID: Bayley Score of Infant Development, MDI: Mental development index, PDI: Psychomotor development index, MRI: Magnetic resonance imaging, CI: Confidence interval, ANOVA: Analysis of variance.

Trial Identifier	Population Studied	Intervention	Ratio N6:N3	AA + DHA Content	LA + ALA + Other LCPUFA Content	Number of Infants	Primary Outcome	Comment
Alshweki et al. (2015) [8]	Eligible preterms were born between 25–32 weeks or <1500g BW. 30% of infants were born <30 weeks GA. Mean data unavailable.	Implementation of dietary N6:N3 ratios of 2:1 (Group A) or 1:1 (Group B) via manipulation of infant formula and monitoring of fatty acid composition of weaning foods. Formula contained fixed DHA (0.3%) and variable AA (0.3%-0.6%). Intervention duration of 12 months.	Group A: 2:1. Group B: 1:1.	Group A = 2:1. Group B = 1:1.	Nil ALA or LA content.	Randomised: 60. 24 vs 21 evaluated at 24 months.	Psychomotor development at 24 months CA. Group A scored more highly than Group B.	Group A scored similarly to breastfed control infants, while Group B scored lower than Group A and breastfed controls.
The DINO Trial (Makrides et al. (2009) [10], Smithers et al. (2010) [11] and Collins et al. (2015) [9])	Preterm infants <33 weeks GA, born across 5 sites in Australia. Median GA at birth 30 weeks.	Randomisation to diets of 'high' DHA content (1%) or 0.3% DHA, achieved by enrichment of breastmilk via maternal oral supplementation, or via DHA supplemented formula.	'High' (1%) DHA group: Formula: 7.7:1, Breast milk: 4.4:1. Standard DHA (0.3%) group: Formula: 9.5:1, Breast milk: 7.4:1.	'High' (1%) DHA group: 0.4:1. Standard DHA (0.3%) group: 1.5:1. Authors reported equal concentrations of AA in both breastmilk and formula (0.6%).	ALA and LA content of feeds was unreported by authors.	Randomised: 667. Evaluated at 19 months: Group A: 332; Group B: 335.	BSID-II at 18 months found no difference in MDI scores between groups.	MDI score interacted with sex and BW: Higher mean MDI in girls and infants born at lower BW (<1250g) at 18 months. Follow up at 26 months CA found no effect of DHA

		Supplementation given until expected term date.						supplementation on visual, language or behavioural development 27, and no benefit to IQ measured at 7 years of age.
Henriksen et al. (2008 [56], 2016 [59]), Almaas et al. (2015 [7], 2016 [58]), Westerberg et al. (2011) [57]	Eligible infants weighed <1500g at birth. Median GA at birth 28.4 weeks.	0.5 mL of a study oil containing 32mg DHA and 31mg AA was added to 100 mL of human milk. Overall DHA and AA content estimated at 58mg and 48mg per 100ml of milk. Oil added from enteral intake of >100 ml/kg/d to discharge.	N6:N3 ratio in milk + oil combination: 5:1 Milk + Control oil combination: 6.6:1	Ratio of AA:DHA in oil = 1:1. Ratio of AA:DHA in milk + oil combination = 0.8:1. No AA/DHA in control oil.	Concentration in milk + oil combination: ALA = 1.32% LA = 13.5% Milk + Control oil: ALA = 1.4% LA = 14.4% Breastmilk: LA = 13% ALA = 1.2%	Randomised: 141. 50 vs 54 analysed at 6 months.	No difference in neurodevelopmental assessments at 6, 18 and 26 months.	Plasma DHA at discharge from hospital associated with MDI scores at 20 months CA.
Clandinin et al. (2005) [60]	Eligible infants were both <36 weeks GA, <1500g BW. Mean GA at birth ~29 weeks.	2 intervention formulae , derived from algal or fish DHA, containing 0.3% DHA and 0.6% AA. Fed within 10 days of starting enteral feed until 12 months CA.	Intervention formulae (algal/fish): Preterm: 7/6.8:1 Post-discharge: 7.4/7.1:1 Term: 9.1/8.6:1 Control: Preterm: 7.8:1 Post-discharge: 8.1:1	2:1	Formulae: LA = 17-18% ALA = 1.5-2.5% Control: LA = 17-19% ALA = 1.6-2.4%	Randomised: 361. 46/59 vs. 54 assessed at 18 months.	Neurodevelopment (BSID-II) using MDI and PDI components at 18 months.	Higher MDI and PDI scores in supplemented groups compared to control, but MDI/PDI scores less than breastfed term infants. Documented that due to minimal exclusion criteria,

			Term: 10.4:1					the study included many infants with concomitant morbidities related to prematurity.
O'Connor et al. (2001) [61]	Eligible infants were born <33wks GA and low/extremely low BW (750-1805g). Multi-country, multi-centre. Mean GA at birth 29.8 weeks.	Two interventional formulae containing DHA and AA derived from fish/fungi or egg/fish. Supplementation began at the start of enteral feeds until 12 months CA.	Intervention (fish/fungi / egg/fish): Preterm: 5.8/6.5:1, Post-discharge: 7.8/8.1:1 Control: Preterm: 6.7:1 Post-discharge: 8:1	Preterm 1.6:1. Post-discharge 2.7:1.	In hospital: fish/fungal= 6.4:1 egg/fish = 7:1 In hospital control: 6.6:1 Postdischarge: fish/fungal= 8.1:1 egg/fish = 8.5:1 Post discharge control: 7.9:1	470 included. Infants evaluated by formula: Fish/fungi: 89/140; Egg/fish: 91/143; Control: 91/144.	BSID score at 12 months. No difference in BSID score at 12 months.	Improved MDI score amongst low birthweight infants in DHA group (<1250g).
Van Wezel-Meijler et al. (2002) [62]	Eligible infants were born <34 weeks GA and weighed <1750g. Mean GA at birth 30.4 weeks.	Formula with or without DHA+AA fed from post-natal day 3-7 until 6 months CA.	Unknown; assumed nil other N3/N6 LCPUFA.	2:1	No reported ALA/LA.	Randomised: 55. Evaluated: 22 vs 20.	MRI at 3 and 12 months. BSID at 3, 6, 9, 12 and 24 months. No differences on MRI or BSID scores at any age.	
Fang et al. (2005) [63]	27 premature infants born 30-37wks GA, weighing >2000g. Mean GA at birth ~33 weeks and birthweight 1.9kg, while the mean gestation at	Standard infant formula compared against the same formula supplemented with 0.05% DHA and 0.10% AA. Supplementation started at full	Total LCPUFA content not included in paper.	2:1	LA:ALA = 10:1.	Randomised: 27. Evaluated at 12 months CA: 15 vs 9.	Visual acuity at 4 and 6 months. BSID at 6 and 12 months CA. Higher MDI and PDI in intervention group.	Small study. Significance by repeated measures ANOVA stated, CI not reported. DHA only 0.05% of total fatty acid content.

	study entrance was 35.5 weeks.	feeds until 6 months CA.						
Fewtrell et al. (2002) [64]	Eligible infants were born <37 weeks GA and weighed <1750g. Mean GA at birth 30.4 weeks and BW 1360g.	DHA (0.17%), AA (0.31%), LA (12%) and ALA (0.7%) supplemented feed versus feed containing LA (10%) and ALA (0.6%) only. Feed provided from 10 days until discharge (mean 33 days.)	Intervention: 11.8:1 Control: 15.3:1	1.8:1 Intervention formula contained 0.5% DHA, 0.04% AA. Control formula was DHA/AA deficient.	Intervention LA:ALA = 17:1, containing 1.5% ALA and 12.3% LA. Control: LA = 16:1.	Randomised: 97. Evaluated: 84 vs 74 at 18 months.	No differences in neurodevelopment measured by BSID-II at 9 and 18 months CA.	Interaction between GA at birth, diet and MDI at 18 months in infants born before 30 weeks.
Fewtrell et al. (2004) [65], Isaacs et al. (2011) [66]	Infants born <3 weeks and weighed <2000g BW. Mean GA at birth 31 weeks.	DHA + GLA in formula from randomization (mean 14 days, range 1–50 days) until 9 months after term.	Intervention: 6.3:1 Control: 7.2:1	1.8:1	Supplement and control formula LA:ALA ratio of ~8:1.	Randomised: 236. Evaluated at 18 months: 106 vs 93.	BSID-II MDI and PDI at 18 months CA. No significant differences between groups. No differences at 9 year follow up (Isaacs et al., 2011).	
Carlson and Werkman (1996) [67]	GA <33 weeks. Mean GA 28 weeks and mean BW 1038g.	DHA + EPA in formula from post-natal day 2–5 until 2 months past term (CA).	Intervention: 8.0:1 Control: 8.8:1	No AA content.	LA 6.5:1 and ALA 7:1 in both control and intervention feeds.	Randomized = 59. Infants evaluated by: Fagan: 15 vs. 12;	Fagan Test of Infant Intelligence administered at 12 months CA. Diet significantly influenced the number of discrete looks.	Only n-3 fatty acids in supplementation. Visual testing result associated by authors as representation of superior information processing capabilities.

Werkman and Carlson (1996) [68]	Eligible infants were born <33 weeks GA.3 Mean GA at birth 29 weeks and BW 1103g. Both studies included infants enrolled at mean 25 days post-natal age.	Formula for preterm and post-discharge periods containing DHA + EPA with no AA. Supplementation until 9 months CA. Mean feeding duration = 50 weeks.	Intervention: Preterm 5.2:1 Post-discharge 6.0:1 Control: Preterm 6.4:1 Post-discharge 6.9:1	No AA content.	LA:ALA 6.5:1 in both control and intervention feeds.	Randomised = 79. Infants evaluated by: Fagan Test = 33 vs 34; BSID-II = 27 vs 27.	Fagan test at 6.5, 9 and 12 months post term found an increased number of discrete looks, increased frequency of looks and reduced look duration in supplemented group. BSID at 12 months CA found no difference.	Results of visual function testing suggested to represent superior visual attention in group supplemented with DHA.
Andrew et al. (2018) (The Dolphin trial) [69]	Eligible infants were born <40 weeks GA with concomitant neonatal comorbidities including; BW <9th centile, intracerebral haemorrhage, white matter injury, moderate-severe hypoxic ischaemic encephalopathy, and/or defined neuroimaging	Supplement containing DHA, EPA, AA, choline, UMP, cytidine monophosphate, vitamin B12, zinc, and iodine added to all feeds from full feeds until 2 years CA.	Supplement N6:N3: ~0.1:1	~0.1:1	No ALA or LA content.	Randomised = 59. 24 vs 19 evaluated.	Cognitive composite score-BSID-III at 12 and 24 months CA.	Poor compliance with supplement. Multiple interventions.

	abnormalities suggestive of brain injury. Mean GA at birth 32.9 weeks and BW 2095g.							
Keim et al. (2018) (The Preemie Tots trial) [70]	Eligible infants were between 18–38 months of age and exhibited early signs of autistic spectrum disorder (ASD) after premature birth <29 weeks GA. Mean 27 months at study enrolment.	Supplement containing N3, N6 and N9 LCPUFA provided between the ages of 18-38 months CA for 90 days.	Intervention: 0.4:1 Control: 2.3:1	GLA:DHA approximately 0.4:1 in intervention supplement.	Intervention 0.4:1 Control 2.3:1	Randomized: 31. All evaluated in ITT analyses.	Parental reported ratings of behaviour and development before and after supplementation using BITSEA scale. Greater improvement in LCPUFA group vs canola oil.	Specific at-risk group. Later intervention, past preterm period, in comparison to other studies.
